# Affective influences without approach-avoidance actions: on the congruence between valence and stimulus-response mappings

**DOI:** 10.3758/s13423-018-1547-1

**Published:** 2018-11-21

**Authors:** Motonori Yamaguchi, Jing Chen

**Affiliations:** 1 Department of Psychology, Edge Hill University, Ormskirk, UK; 2 Department of Psychology, Old Dominion University, Norfolk, VA USA

**Keywords:** Stimulus–response compatibility, Affective valence, Mixed mapping, Response selection, Task representation

## Abstract

The valence of stimuli can influence performance in the spatial stimulus–response compatibility task, but this observation could arise from the process of selecting responses or selecting stimulus–response mappings. The response-selection account proposes that spatial compatible and incompatible keypress responses serve as approaching and avoiding actions to a valenced target. The mapping-selection account suggests that there is congruence between stimulus valence and stimulus–response mappings; positive-compatible/negative-incompatible is more congruent than negative-compatible/positive-incompatible. Whereas affective valence was part of the target stimuli to which participants responded in previous studies, the present study isolated affective valence from the target by presenting an additional mapping cue separately from the target, so that spatially compatible and incompatible keypress responses could no longer serve as approaching and avoiding actions to valenced target stimuli. The present results revealed that responses were still faster when positive and negative mapping cues were assigned to the spatially compatible and incompatible mappings than when the assignment was reversed. The finding supports the mapping-selection account, indicating that positive and negative cues influence performance without approach–avoidance actions to valenced stimuli. The experiment provides important implications as to how tasks are represented and are dependent on affective processing.

In choice-reaction tasks, responses are faster when the locations of stimulus and response correspond than when they do not (Fitts & Seeger, 1953). The influence of this spatial stimulus–response compatibility (SRC) is prevalent in operational settings, such as driving a car (Müsseler, Aschersleben, Arning, & Proctor, 2009; Sabic & Chen, 2017) and piloting an aircraft (Yamaguchi & Proctor, 2006). Although automated systems are quickly developing, human operations are still critical in maintaining safety in complex environments. One of the unique features of human operations is that they are subject to emotional reactions. Negative emotions can lead to annoyance, hazardous behaviors, and then, fatal accidents (e.g., Wells-Parker et al., 2002). Although emotion has been of central importance in understanding and predicting human behaviors, much is remained to be understood about its relationship to human cognitive performance. Many studies investigated the influences of affective valence, a component of emotion (Russell, 2003), on manual actions (e.g., Chen & Bargh, 1999; Eder & Rothermund, 2010; Solarz, 1960) or verbal responses (De Houwer, Crombez, Baeyens, & Hermans, 2001), but only few examined the influences of affective processing on such fundamental performance parameters as spatial SRC (Conde et al., 2011; Yamaguchi Chen, Mishler, & Proctor 2018). The purpose of the present study was to extend the understanding of how affective valence influences performance in a spatial SRC task.

Affective processing is known to influence various types of cognitive processes, such as attention (Öhman, Flykt, & Esteves, 2001; Yamaguchi & Harwood, 2017), memory (Ayçiçeǧi & Harris, 2004), and execution of manual responses (Chen & Bargh, 1999; Solarz, 1960). Recently, it has also been suggested that affective valence of stimuli modulates the spatial SRC effect (Conde et al., 2011). In a typical spatial SRC task, participants respond to stimuli that appear on the left or right of the fixation on a display by pressing left and right response keys. There are two different experimental blocks, in which participants are given different task instructions, or stimulus–response (S–R) mappings. In the *compatible mapping* block, participants respond to stimuli by pressing keys whose locations correspond to the stimulus locations (i.e., pressing the left key to stimuli on the left and the right key to stimuli on the right). In the *incompatible mapping* block, participants respond to stimuli by pressing keys whose locations do not correspond to the stimulus locations (i.e., pressing the left key to stimuli on the right and the right key to stimuli on the left). Responses are faster with the compatible mapping than with the incompatible mapping. This is known as the *spatial SRC effect*.

Conde et al. (2011) first reported a finding that affective valence of the target stimuli to which participants responded modulated the SRC effect. In their study, the targets were avatars that wore the uniform of participants’ favorite soccer team (positive stimuli) or that of the rival team (negative stimuli). In a block of trials, participants responded to positive stimuli by pressing spatially compatible response keys, but to negative stimuli by pressing spatially incompatible response keys (*positive-compatible/negative-incompatible assignment*); in another block, the assignment of avatars’ uniforms to compatible and incompatible mappings was reversed (*negative-compatible/positive-incompatible assignment*). The researchers compared spatially compatible and incompatible trials for positive and negative stimuli separately, and found a standard SRC effect for positive stimuli but a reversed SRC effect (favoring spatially noncorresponding responses to the stimulus locations) for negative stimuli (see also Cavallet et al., 2016, a replication with ADHD patients). The researchers proposed that pressing keys that correspond to the stimulus location is equivalent to “approaching” actions toward the stimuli, whereas pressing keys that do not correspond to the stimulus location is equivalent to “avoiding” actions. Consequently, they suggested that the reversed SRC effect was due to avoiding negative stimuli being more congruent than approaching these stimuli. This explanation would mean that the congruence of approach and avoidance actions with positive and negative stimuli, respectively, outweighed spatial SRC. We call this explanation the *response-selection account*.

Proctor (2013) pointed out that Conde et al. (2011) used an experimental setting known as a *mixed-mapping condition* in the SRC literature (Shaffer, 1965; Yamaguchi & Proctor, 2006). With mixed mappings, spatial SRC effect is typically reduced substantially (Yamaguchi & Proctor, 2006), eliminated completely (Shaffer, 1965), or reversed to favor incompatible stimulus–response pairs (Proctor, Yamaguchi, Dutt, & Gonzalez, 2013), even when no valence cue is involved in the task. In fact, Conde et al. compared compatible and incompatible trials from different cue-mapping assignments, but when the same data were assessed for the same cue-mapping assignments, there was little evidence of the SRC effect, consistent with the findings in the previous mixed-mapping studies. Instead, responses appeared generally faster for the positive-compatible/negative-incompatible assignment than for the negative-compatible/positive-incompatible assignment. Thus, it appeared that when positive and negative cues were assigned, respectively, to spatially compatible and incompatible mappings, these mappings were retrieved faster than when positive and negative cues were assigned, respectively, to spatially incompatible and compatible mappings. We call this explanation the *mapping-selection account*.

These two competing accounts were tested previously (Yamaguchi et al., 2018), in which the data were analyzed in both Conde et al.’s (2011) and Proctor’s (2013) manners. The study replicated the influence of stimulus valence on performance of the SRC task as in Conde et al.’s analysis. However, the study also confirmed that with Proctor’s analysis, the SRC effect was absent with mixed mappings, but responses were generally faster for the positive-compatible/negative-incompatible assignment than for the negative-compatible/positive-incompatible assignment. The study also revealed that there was little influence of stimulus valence on the spatial SRC effect in a similar task setting, known as the Simon task (Simon & Rudell, 1967), in which participants selected responses based on stimulus valence, not spatial S–R mappings. This result contradicted the response-selection account because it predicted that the SRC effect should still be reversed for negative stimuli in the Simon task. The results were consistent with the mapping-selection account, as it predicted no influence of stimulus valence when the task does not involve mapping selection.

The purpose of the present study was to further distinguish the response-selection and mapping-selection accounts. In both Conde et al.’s (2011) and Yamaguchi et al.’s (2018, Experiment 1) studies, participants responded to target stimuli that contained either a positive or negative value, and spatially compatible and incompatible responses to the targets could be interpreted as approach and avoidance actions to the targets, respectively. In the present study, we separated stimulus valence from the targets by presenting separate mapping cues that were either positive (flowers) or negative (spiders), and the cue valence indicates whether to respond compatibly or incompatibly to target locations. The mapping cue always appeared in the screen center, and responses were left and right keypresses; consequently, there was no approach or avoidance action to the valenced mapping cue. Similarly, the targets occurred on the left or right, but they were always blue rectangles that were valence neutral; hence, spatially compatible and incompatible responses to the targets could not be interpreted as approach and avoidance actions to valenced stimuli. Therefore, the response-selection account would predict little influence of the valence of mapping cues when the targets are valence neutral, as in the present condition. The mapping-selection account would still predict that spatially compatible and incompatible mappings are retrieved faster by positive and negative mapping cues, respectively; thus, responses should be faster when flowers and spiders were assigned to the compatible and incompatible mappings, respectively, than when the mapping assignment was reversed.

## Method

### Participants

Forty eight participants (43 female; mean age = 20.7 years, *SD* = 4.3 years) at Old Dominion University participated for partial credits toward their psychology courses.^1^ The protocol was approved by the Institutional Review Board of Old Dominion University. Informed consent was obtained from all participants.

### Apparatus and stimuli

The apparatus consisted of a 19-in. LCD monitor and a personal computer. Participants wore noise-canceling headphones, which presented auditory feedback. The target stimuli were blue rectangles (4.3 cm × 2.3 cm) that appeared on the left or right side of the screen, with a center-to-center distance of 34.3 cm. The mapping cues were photographs of 10 flowers and 10 spiders (14-cm wide × 8-cm high), which were used in our previous study (see Yamaguchi et al., 2018). The cues appeared at the screen center. Responses were registered by pressing the “z” and “/” keys on a QWERTY keyboard.

### Procedure

Participants sat in front of the monitor at the distance of approximately 60 cm, placed their left and right index fingers on the response keys, and were instructed to respond to blue rectangles (targets) as quickly and as accurately as possible. There were two phases with different cue-mapping assignments for each participant. One phase required participants to make spatially compatible responses to the target when the mapping cue was a flower, and spatially incompatible responses when the mapping cue was a spider (flower-compatible/spider-incompatible assignment). The other phase required participants to make spatially compatible responses to the target when the mapping cue was a spider, and spatially incompatible responses when the mapping cue was a flower (spider-compatible/flower-incompatible assignment). The order of the two phases was counterbalanced across participants. Also, within each phase, the interval between the mapping cue and the target (stimulus onset asynchrony, or SOA) was manipulated between blocks. In one block, the SOA was 0 ms; the mapping cue and the target appeared simultaneously. In the other block, the SOA was 500 ms; the target appeared 500 ms after onset of the mapping cue. Each phase started with one block of 16 practice trials and two blocks of 120 test trials each; one test block used the 0-ms SOA and the other the 500-ms SOA. The order of the two SOA blocks was counterbalanced across participants and maintained between the two phases for a given participant.

Each trial started with a fixation mark at the screen center for 500 ms, replaced by a mapping cue (spider or flower). The target (blue rectangle) followed the mapping cue with an SOA. Both the target and the cue stayed on the screen until a response key was pressed. The target appeared on the left side of the display in half of the trials and on the right side in the other half, and the order of the locations was randomly determined. For each of the 20 task cues, the two target locations occurred equally frequently. These task cues also occurred equally frequently within a block. A response was followed by the message “Correct” or “Incorrect,” which stayed on the screen for 1,000 ms. A 500-Hz tone occurred for an incorrect response. Response time (RT) was the interval between target onset and a keypress.

## Results

**Fig. 1:**
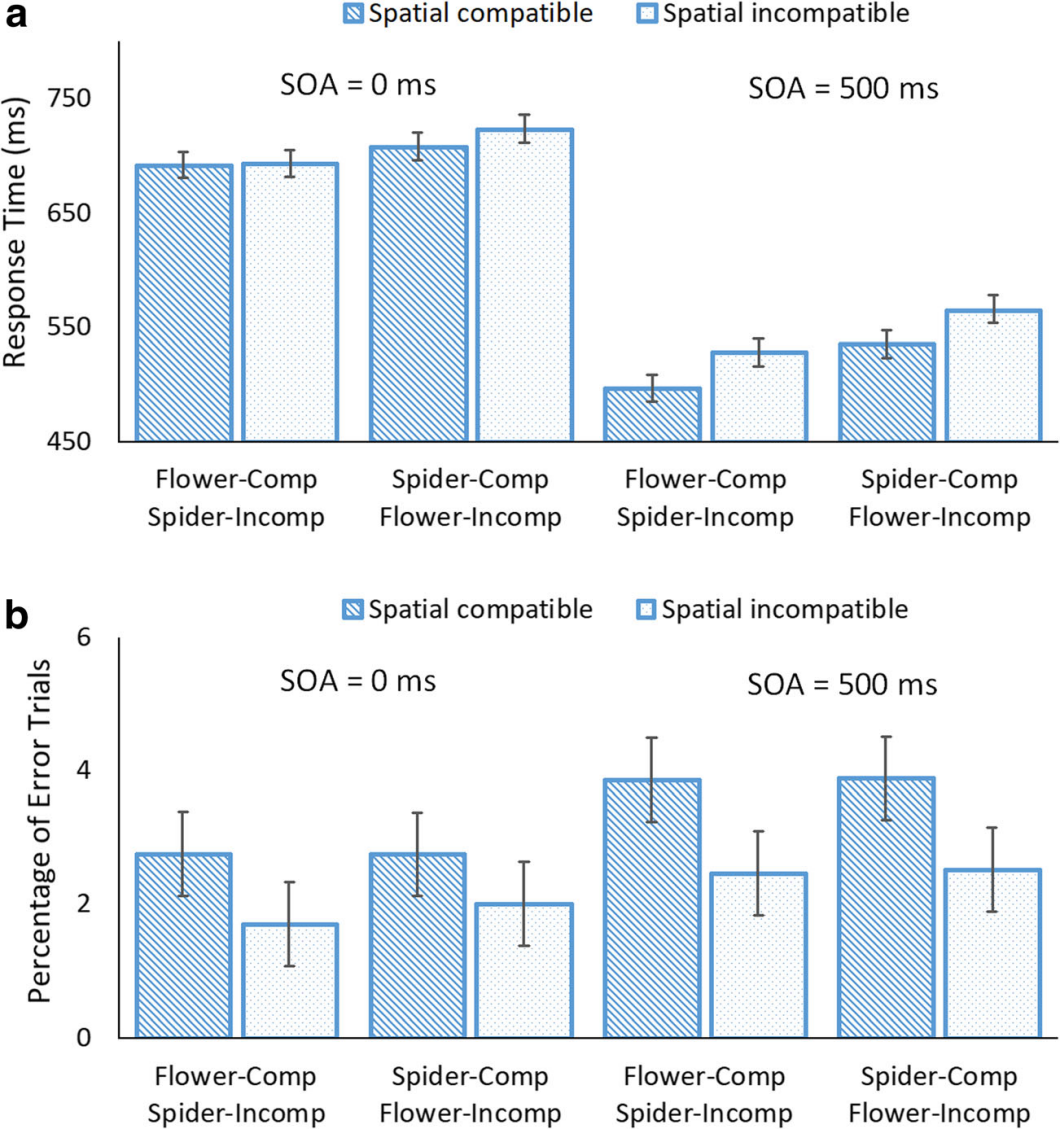
Mean response time (**a**) and percentage of error trials (**b**) for spatially compatible and incompatible trials as a function of SOA (0 ms vs. 500 ms) and cue-mapping assignment (flower-compatible/spider-incompatible vs. spider-compatible/flower-incompatible). Error bars are 95% within-subject confidence intervals around the means (Loftus & Masson, 1994)

Trials were discarded if RT was shorter than 200 ms or longer than 3,000 ms (.55% of all trials). Mean RT for correct responses and percentage of error trials (PE) were computed for each participant. One participant exceeded 10% error rate and was excluded from the analysis. RT and PE of the remaining participants are summarized in Fig. 1.

Note that the data could be analyzed in two ways, one based on Conde et al.’s (2011) and the other based on Proctor’s (2013) analyses. In the first analysis, the data are to be submitted to 2 (cue valence: positive vs. negative) × 2 (spatial compatibility: compatible vs. incompatible) × 2 (SOA: 0 ms vs. 500 ms) ANOVAs. In the second analysis, the data are to be submitted to 2 (cue-mapping assignment: flower-compatible/spider-incompatible vs. spider-compatible/flower-incompatible) × 2 (spatial compatibility: compatible vs. incompatible) × 2 (SOA: 0 ms vs. 500 ms) ANOVAs. However, these analyses only differ in how the factors are combined, with the interaction between cue valence and spatial compatibility in Conde et al.’s analysis corresponding to the main effect of cue-mapping assignment in Proctor’s analysis (see Yamaguchi et al., 2018). We carried out the second analysis because the interpretations are more straightforward. The results are summarized in Table 1.

**Table 1:** ANOVA results

Factor	*df*	*MSE*	*F*	*p*	η_p_^2^
*Response time*					
**Cue-mapping assignment (CMA)**	**1, 46**	**16,543.65**	**5.23**	**.027**	**.102**
**SOA**	**1, 46**	**11,953.24**	**234.51**	**<.001**	**.836**
**Spatial compatibility (SC)**	**1, 46**	**2,206.94**	**16.07**	**<.001**	**.259**
CMA × SOA	1, 46	7,656.84	<1	.430	.014
CMA × SC	1, 46	2,144.81	<1	.503	.010
**SOA × SC**	**1, 46**	**1,568.28**	**7.96**	**.007**	**.147**
CMA × SOA × SC	1, 46	166.83	<1	.375	.017
*Percentage of errors*					
CMA	1, 46	6.60	<1	.722	.003
**SOA**	**1, 46**	**8.29**	**8.77**	**.005**	**.160**
**SC**	**1, 46**	**6.75**	**18.13**	**<.001**	**.283**
CMA × SOA	1, 46	4.16	<1	.797	.001
CMA × SC	1, 46	3.65	<1	.672	.004
SOA × SC	1, 46	5.43	<1	.328	.021
CMA × SOA × SC	1, 46	4.59	<1	.737	.002

*Note.* Bold indicates significance at alpha = .05.

The critical effect that distinguishes the response-selection and mapping-selection accounts is the main effect of cue-mapping assignment in the current analysis (which is the same as the interaction between cue valence and spatial compatibility in Conde et al.’s, 2011). According to the response-selection account, this effect depends on responding actions being approaching or avoiding valenced targets. Given that there were no approach–avoidance actions to valenced targets in the present task setting, the response-selection account would predict a null effect. According to the mapping-selection account, there should be a significant main effect of cue-mapping assignment because it depends on whether positive and negative stimuli are assigned to congruent spatial S–R mappings (i.e., responses should be faster for the flower-compatible/spider-incompatible assignment than the spider-compatible/flower-incompatible assignment). As in previous studies, the main focus was this effect in RT, but we also report the same analysis on PE.

### Response time

For RT, the main effect of cue-mapping assignment was significant, and it did not interact with other factors. Responses were faster with the flower-compatible/spider-incompatible assignment (*M* = 602 ms) than with the spider-compatible/flower-incompatible assignment (*M* = 632 ms), yielding an overall advantage of 30 ms for the flower-compatible/spider-incompatible assignment over the opposite assignment. There was also a significant interaction between SOA and spatial compatibility, indicating that the SRC effect depended on SOA; the SRC effect was 31 ms for longer SOA but was 8 ms for shorter SOA. The former effect was significant, *t*(46) = 5.72, *p* < .001, Cohen’s *d* = .834, but the latter was not, *t*(46) = 1.11, *p* = .274, Cohen’s *d* = .161. There were main effects of SOA and spatial compatibility. Responses were faster overall for longer SOA (*M* = 531 ms) than for shorter SOA (*M* = 703 ms), and for spatially compatible trials (*M* = 607 ms) than for spatially incompatible trials (*M* = 627 ms), yielding a 20-ms SRC effect. All significant factors produced large effect sizes (see Table 1).

### Percentage of error trials

For PE, there were main effects of SOA and spatial compatibility. Reponses were more accurate for shorter SOA (*M* = 2.30%) than for longer SOA (*M* = 3.18%), and for spatially incompatible trials (*M* = 2.17%) than for spatially compatible trials (*M* = 3.31%), reversing the spatial SRC effect. Both effects indicated large effect sizes (see Table 1). No other effects were significant.

## Discussion

The present experiment investigated the influence of affective valence in the spatial SRC task (Conde et al., 2011; Yamaguchi et al., 2018). To isolate spatial SRC from the effect of approach/avoidance actions to targets, affective valence was now presented within the mapping cues that were separate from the target. The targets were valence-neutral (blue rectangles), and spatially compatible and incompatible keypresses to the targets could not be considered to be approach or avoidance actions to valenced stimuli. The results revealed that responses were still faster for the flower-compatible/spider-incompatible assignment than for the spider-compatible/flower-incompatible assignment. This advantage of the former assignment indicates that the assignment of positive and negative cues to the spatially compatible and incompatible S–R mappings is more congruent than the reversed cue-mapping assignment (Proctor, 2013; Yamaguchi et al., 2018), consistent with the mapping-selection account. The results indicate that the retrieval of these mapping rules depended on the valence of the mapping cues.

The separate mapping cue from the targets allowed a manipulation of the SOA between the mapping cue and the target. Shaffer (1965) found that the SRC effect was obtained when there was a temporal gap between the mapping cue and the target (SOA = 333 ms), but it disappeared when there was no gap (SOA = 0 ms). In the previous studies (Conde et al., 2011; Yamaguchi et al., 2018), the valence was a part of the target attribute, so there was no temporal gap, eliminating the SRC effect. The present experiment replicated Shaffer’s finding, showing a significant SRC effect only for 500 ms but not for 0 ms. Therefore, the absence of the SRC effect with mixed mappings in the previous studies was not a necessary condition for the cue-mapping assignment to influence performance in the SRC task. The cue-mapping assignment did not affect the SRC effect, implying that valence influenced a process other than response selection from which the SRC effect emerges (Hommel, 1995). The study also showed that the SRC effect in PE was reversed overall, which was also found in previous studies (e.g., Proctor et al., 2013; Yamaguchi et al., 2018; Yamaguchi & Proctor, 2006). This suggests overcompensation of the natural tendency to respond to spatial stimuli compatibly, but the cue-mapping assignment did not influence the outcome, either.

Note that the response-selection account and the mapping-selection account are not mutually exclusive, and a previous study demonstrated that affective valence of the target could influence response selection in some cases (i.e., when responses are made by a joystick with a moving cursor on the monitor; Yamaguchi et al., 2018). Yet the present results are clear-cut as to the conclusion that affective valence could influence mapping selection even with keypress responses. The exact mechanism behind the affective influence on mapping selection should be an issue to be explored in future investigations. It may be a correspondence between the polarities of cues and S–R mappings (Proctor, 2013). This suggestion seems to be consistent with the neurocognitive theory of cognitive control (e.g., Botvinick, 2007), according to which response conflict on incompatible trials triggers an aversive signal from the anterior prefrontal cortex that strengthens cognitive control. The idea of conflict as an aversive signal was supported in an affective priming task (Dreisbach & Fischer, 2012), in which incongruent Stroop stimuli facilitated an evaluation of negatively valenced pictures. In the present experiment, the incompatible S–R mapping may be received as a negative affective event and is congruent with negative cues. Just as S–R compatibility facilitates retrieval of a responses, the cue-mapping congruence would facilitate retrieval of the mapping. At this stage of investigation, this explanation seems to be most plausible for the affective influence in the SRC task.

The present finding is important because it suggests that factors affecting response selection could also extend to other types of selection or decision-making processes involved in more complex task environments. Cognitive processes underlying complex tasks are structured hierarchically and involve series of selection processes (e.g., Cooper & Shallice, 2006). As hierarchical theories would imply, different levels of selection processes operate independently and can be sensitive to different environmental factors. Previous studies have focused mostly on influences of affective valence on action selection or execution (e.g., Chen & Bargh, 1999; De Houwer et al., 2001; Eder & Rothermund, 2010; Solarz, 1960), but the present study implies that affective valence is also relevant to higher-level selection processes. Because everyday activities are typically complex and involves hierarchical structures, it would be interesting to see how the present results could generalize to real-world operations in future studies. The present study supported the generalizability of the phenomena across different types of valended stimuli (Conde et al., 2011; Yamaguchi et al., 2018), and we have no reason to believe that the results depend on other characteristics of the participants, materials, or context. As the statement of constraints of generality (Simons, Shoda, & Lindsay, 2017, p. 1126), however, we postulate that the boundary conditions of the present results are still unknown and have to be explored in future investigations.

### Author note

The raw data can be retrieved from the Open Science Framework project page (https://osf.io/nvrx3/). We thank Kyle Brinn and Khaila Taylor for their help on data collection.

## Footnotes

1 With all variables being within-subject factors, the present sample size would result in a statistical power of greater than .99 for a medium effect size at alpha = .05, assuming the correlation of .8 between the measures.

## References

[CR1] Ayçiçeǧi A, Harris CL (2004). Bilinguals’ recall and recognition of emotion words. Cognition and Emotion.

[CR2] Botvinick MM (2007). Conflict monitoring and decision making: Reconciling two perspectives on anterior cingulate function. Cognitive, Affective, & Behavioral Neuroscience.

[CR3] Cavallet M, Chaim-Avancini TM, Biazoli CE, Bazán PR, de Silva MA, Cunha PJ, Gawryszewski LG (2016). Influence of emotional stimulus valence on inhibitory control in adults with and without ADHD. Experimental Brain Research.

[CR4] Chen M, Bargh JA (1999). Consequences of automatic evaluation: Immediate behavioral predispositions to approach or avoid the stimulus. Personality and Social Psychology Bulletin.

[CR5] Conde EFQ, Jazenko F, Fraga-Filho RS, da Costa DH, Torro-Alves N, Cavallet M, Gawryszewski LG (2011). Stimulus affective valence reverses spatial compatibility effect. Psychology & Neuroscience.

[CR6] Cooper RP, Shallice T (2006). Hierarchical schemas and goals in the control of sequential behaviour. Psychological Review.

[CR7] De Houwer J, Crombez G, Baeyens F, Hermans D (2001). On the generality of the affective Simon effect. Cognition and Emotion.

[CR8] Dreisbach G, Fischer R (2012). Conflicts as aversive signals. Brain and Cognition.

[CR9] Eder AB, Rothermund K (2010). Automatic influence of arousal information on evaluative processing: Valence-arousal interactions in an affective Simon task. Cognition and Emotion.

[CR10] Fitts PM, Seeger CM (1953). S-R compatibility: Spatial characteristics of stimulus and response codes. Journal of Experimental Psychology.

[CR11] Hommel, B. (1995). Stimulus-response compatibility and the Simon effect: Toward an empirical clarification. *Journal of Experimental Psychology: Human Perception and Performance, 21*, 764–775 10.1037//0096-1523.17.1.246 1826315

[CR12] Loftus GR, Masson MEJ (1994). Using confidence intervals in within-subject designs. Psychonomic Bulletin & Review.

[CR13] Müsseler J, Aschersleben G, Arning K, Proctor RW (2009). Reversed effects of spatial compatibility in natural scenes. American Journal of Psychology.

[CR14] Öhman A, Flykt A, Esteves F (2001). Emotion drives attention: Detecting the snake in the grass. Journal of Experimental Psychology: General.

[CR15] Proctor RW (2013). Stimulus affect valence may influence mapping-rule selection but does not reverse the spatial compatibility effect: Reinterpretation of Conde et al. (2011). Psychology & Neuroscience.

[CR16] Proctor RW, Yamaguchi M, Dutt V, Gonzalez C (2013). Dissociation of S-R compatibility and Simon effects with mixed tasks and mappings. Journal of Experimental Psychology: Human Perception and Performance.

[CR17] Russell JA (2003). Core affect and the psychological construction of emotion. Psychological Review.

[CR18] Shaffer LH (1965). Choice reaction with variable S-R mapping. Journal of Experimental Psychology.

[CR19] Simon JR, Rudell AP (1967). Auditory S-R compatibility: The effect of an irrelevant cue on information processing. Journal of Applied Psychology.

[CR20] Simons DJ, Shoda Y, Lindsay DS (2017). Constraints on Generality (COG): A proposed addition to all empirical papers. Perspectives on Psychological Science.

[CR21] Solarz AK (1960). Latency on instrumental responses as a function of compatibility with the meaning of eliciting verbal signs. Journal of Experimental Psychology.

[CR22] Sabic, E. & Chen, J. (2017). Left or right: Auditory collision warnings for driving assistance systems. In *Proceedings of the Human Factors and Ergonomics Society 61st international annual meeting*. Washington, DC: HFES.

[CR23] Wells-Parker, E., Ceminsky, J., Hallberg, V., Snow, R. W., Dunaway, G., Guiling, S., . . . Anderson, B. (2002). An exploratory study of the relationship between road rage and crash experience in a representative sample of US drivers. *Accident, Analysis, and Prevention*, *34*, 271–278. 10.1016/s0001-4575(01)00021-5 11939355

[CR24] Yamaguchi M, Harwood SL (2017). Threat captures attention but does not affect learning of contextual regularities. Cognition and Emotion.

[CR25] Yamaguchi M, Proctor RW (2006). Stimulus-response compatibility with pure and mixed mappings in a flight task environment. Journal of Experimental Psychology: Applied.

[CR26] Yamaguchi, M., Chen, J., Mishler, S., & Proctor, R. W. (2018). Flowers and spiders in spatial stimulus-response compatibility: Does affective valence influence selection of task-sets or selection of responses? *Cognition and Emotion, 32*(5), 1003–1017. 10.1080/02699931.2017.1381073. 10.1080/02699931.2017.1381073 28946804

